# Gaming disorder: Prevalence and association with psychosocial outcomes in the German general adult population

**DOI:** 10.1016/j.abrep.2026.100682

**Published:** 2026-02-21

**Authors:** André Hajek, Andrew Stickley, Karl Peltzer, Supa Pengpid, Hans-Helmut König

**Affiliations:** a Department of Health Economics and Health Services Research, University Medical Center Hamburg-Eppendorf, Hamburg Center for Health Economics, Hamburg, Germany; b Department of Preventive Intervention for Psychiatric Disorders, National Institute of Mental Health, National Center of Neurology and Psychiatry, 4-1-1 Ogawahigashi-cho, Kodaira, Tokyo 187-8553, Japan; c Department of Health Education and Behavioral Sciences, Faculty of Public Health, Mahidol University, Bangkok, Thailand; d Department of Psychology, University of the Free State, Bloemfontein, South Africa; e Department of Medical Research, China Medical University Hospital, China Medical University, Taichung, Taiwan; f Department of Public Health, Sefako Makgatho Health Sciences University, Pretoria, South Africa; g Department of Healthcare Administration, College of Medical and Health Science, Asia University, Taichung, Taiwan

**Keywords:** Gaming disorder, Loneliness, Social isolation, Depression, Anxiety, Social withdrawal, Mental health, Gaming, Life satisfaction, Hikikomori, Satisfaction with life

## Abstract

Aim: To identify the prevalence of gaming disorder and its association with psychosocial outcomes (including mental health, subjective well-being and social disconnectedness) in the general adult population in Germany and whether these associations differ by gender and/or age. Methods: Individuals aged 18 to 74 years (mean age: 47 years, SD: 15 years) were surveyed (quota sample of the general adult German population in January 2025, n = 3,270). Psychometrically validated tools were used to quantify the outcomes. The Internet Gaming Disorder Scale was used to quantify the problematic use of computer games (cut-off of 5 or higher to indicate probable gaming disorder). Results: Overall, 59.2% of the individuals in the sample played computer games (offline or online). Among these, 11.9% had a probable gaming disorder, equaling a prevalence of 7% in the entire sample. In adjusted regression analyses, individuals playing computer games, but without a probable gaming disorder, had significantly poorer psychosocial outcomes in terms of depressive symptoms, anxiety symptoms, social isolation, social withdrawal and poor life satisfaction compared to those not playing computer games. Individuals with a probable gaming disorder had markedly poorer psychosocial outcomes compared to their counterparts who did not play computer games. Comparable findings were observed in gender- and age-stratified analyses. Conclusions: In this study, 7% of the sample had a probable gaming disorder. Analyses indicated that such individuals are at high risk of poorer psychosocial outcomes and that these associations are observed across gender and age groups. Future longitudinal studies are recommended to further elucidate these associations.

## Highlights

• Limited knowledge exists regarding gaming disorder in the general adult population.
• Based on the general German adult population, 7% had a probable gaming disorder.
• Gaming disorder was markedly associated with poor psychosocial outcomes.
• Comparable findings were observed in gender- and age-stratified analyses.
• This is helpful to address individuals at risk of poor psychosocial outcomes.

## 1 Introduction

In recent years, increasing attention has been focused on the phenomenon of video game playing and the problems associated with it. Internet Gaming Disorder (IGD) has been included in the most recent (fifth) edition of the Diagnostic and Statistical Manual of Mental Disorders (American Psychiatric Association, 2013), while Gaming Disorder (GD) is also officially listed in the 11th Revision of the International Classification of Diseases (ICD) (World Health Organization, 2025). IGD or GD (which are commonly used interchangeably) both refer to the problematic use of online or offline video games. A growing body of research has indicated that problematic video game use represents a significant public health issue (Rumpf et al., 2018) that is associated with a variety of negative outcomes. For example, a previous systematic review showed an association between problem gaming and suicidal ideation (Erevik et al., 2022), while a scoping review (Richard et al., 2020) also showed that GD can contribute to poorer mental health, decreases in satisfaction with life, and a deterioration in academic performance. It can also have negative consequences for romantic relationships (Kosa et al., 2025). Furthermore, it is positively associated with job stress (Cao et al., 2022).

A previous study (Wartberg et al., 2017) reported that the estimated prevalence of IGD among individuals aged 12 to 25 years in Germany was 5.7% (95% CI: 4.5% to 6.9%) in September 2016 (based on the Internet Gaming Disorder Scale). It further found that IGD was significantly associated with, among other things, higher depressive and anxiety symptoms and more frequent neglect of social contacts (Wartberg et al., 2017). Moving beyond Germany, a recent meta-analysis that used data from 227,665 individuals in 29 countries revealed an overall pooled prevalence of GD of 3.3% (95% CI: 2.6% to 4.0%) (Kim et al., 2022). Similar figures were identified in another recent global meta-analysis (Stevens et al., 2021). However, it was noticeable that the mean age of individuals in the included studies was 19.9 years (SD: 9.5 years) (Kim et al., 2022), and that most of the studies focused on adolescent samples (Kim et al., 2022). Indeed, in only two of the included studies was the mean age of individuals above 40 years old: one Dutch study where the data collection occurred in 2009 (Haagsma et al., 2012) and one study of Canadian adults with data collection in 2015 (Sanders et al., 2017).

Including individuals aged 40 years and over in research on GD is warranted because they will have grown up with computer games (e.g., in the 1970s or 1980s). Moreover, given the age of previous studies that included adults (Kim et al., 2022), the use of up-to-date data is essential for various reasons. For example, the gaming industry and what constitutes gaming have changed considerably in the past 10 to 15 years, partly because an increasing number of people are also playing on mobile devices (such as tablets or smartphones) (Yi et al., 2019). Moreover, the potential impact of various events in recent years (such as the COVID-19 pandemic, military conflicts in Eastern Europe and the Near East etc.,) and the fact that the difficulty in coping with such events may have contributed to both GD and the negative outcomes associated with it (Putra et al., 2023, Teng et al., 2021), also necessitates the use of more recent data on GD and its potential consequences.

Overall, while gaming disorder has been extensively studied among adolescents and young adults (Kim et al., 2022), far less is known about the prevalence of GD in samples of the general adult population (that include *middle-aged and older adults*) or about the association of GD with a more comprehensive set of *psychosocial outcomes.* However, such knowledge is essential when it comes to identifying individuals at risk of poorer psychosocial outcomes, especially given the fact that such outcomes have been associated with, among other things, morbidity and mortality (Deason et al., 2025; Hajek, Sutin, et al., 2025). To address these deficits, the current study aimed to determine the prevalence of GD in the general German adult population (and also in important subgroups) and to examine the association of GD with a comprehensive set of psychosocial outcomes (relating to mental health, subjective well-being, and social disconnectedness) in this population in Germany.

## 2 Methods

***Sample***.

The data for this study were obtained through an online survey, which was conducted in January 2025. The survey was conducted by Bilendi (a highly reputable and ISO-certified market research company). To achieve a sample representative of the German adult population, a quota sampling method was employed (cross-quota for age and gender, and uncrossed quota for federal state). The selected sample consisted of 3,270 individuals aged between 18 and 74 years old. Among the total sample, the mean age was 47.0 years (SD: 15.3 years, ranging from 18 to 74 years), with 50.4% of the sample being female.

All participants provided informed consent, and the study received approval from the Local Psychological Ethics Committee at the University Medical Center Hamburg-Eppendorf (LPEK-0849).

***Psychosocial outcomes***.

The Patient Health Questionnaire-9 (PHQ-9, consisting of nine items) (Kroenke et al., 2001) was used to assess depressive symptoms in the past two weeks. The sum score ranges from 0 to 27 (with higher scores reflecting more depressive symptoms). The German version has sound psychometric properties (Kliem et al., 2024). The internal validity was high – Cronbach’s alpha was 0.90.

The occurrence of anxiety symptoms in the past two weeks was assessed with the seven-item Generalized Anxiety Disorder-7 (GAD-7) scale (Spitzer et al., 2006). The summed score ranges from 0 to 21, with higher values reflecting more anxiety symptoms. The German version has favorable psychometric characteristics (Kliem et al., 2025). Cronbach’s alpha for this measure was 0.92 in the current study.

The six-item De Jong Gierveld loneliness scale (Gierveld & Tilburg, 2006) was used to assess loneliness levels. An overall score is obtained by averaging all items. This score ranges from 0 to 6, with higher values reflecting greater levels of loneliness. Favorable psychometric properties have been reported for the German version (De Jong Gierveld & Van Tilburg, 2010) Cronbach’s alpha was 0.78 for this measure.

The Bude and Lantermann tool (consisting of four items) (Bude & Lantermann, 2006) was used to measure perceived social isolation. By averaging all four items, a total score was calculated. This score varies from 1 to 4, with higher scores corresponding to higher perceived social isolation levels. Cronbach’s alpha was 0.91 for this measure in this study. This has also been demonstrated before for the German version (Bude & Lantermann, 2006).

The 25-item Hikikomori Questionnaire (HQ-25) (Teo et al., 2018) was used to measure social withdrawal. The German version has excellent psychometric properties (Hajek et al., 2024). By summing up all of its items, a sum score is obtained, which ranges from 0 to 100, with higher scores reflecting greater social withdrawal (Cronbach's alpha was 0.92).

Life satisfaction was assessed with the Satisfaction with Life Scale (Diener et al., 1985), which consists of five items, which are summed to generate a total score that ranges between 5 and 35, with higher values corresponding to higher life satisfaction levels. Cronbach’s alpha was 0.90 in this study. Good psychometric properties for the German version have also been demonstrated in previous research (Hinz et al., 2018).

***Gaming disorder***.

The validated German version (Jeromin et al., 2016) of the Internet Gaming Disorder Scale (IGDS) which was developed by Lemmens et al. (2015) was used to quantify the problematic use of computer games. It is worth noting that the IGDS items refer to both games on a computer/laptop or a game console and those played on other types of devices (e.g., a mobile phone, tablet, etc.), whether online and/or offline during the past 12 months. Items include: “During the last year, have there been hour-long periods when all you can think of is the next time you can play a game?” or “During the last year, have you played games so that you would not have to think about unpleasant things?”. Sound psychometric properties of the German version have been shown (Jeromin et al., 2016). Cronbach’s alpha was 0.85 in this study.

Individuals respond with no (0) or yes (1) answers to each of the nine items. The resulting summed score ranges from 0 to 9, whereby higher values reflect the more problematic use of computer games. The established cut-off of 5 or higher (Lemmens et al., 2015) (DSM-V approach) was used to indicate probable GD.

***Covariates***.

Informed by previous research (Hajek, 2013, Hajek et al., 2022; Hajek, Zwar, et al., 2025), a number of covariates were included in the regression analyses. Sociodemographic factors included age, gender (male; female; diverse), federal state, education (ISCED-97 classification (UNESCO, 2006): low, medium, high), marital status (single; divorced; widowed; living together: married or in a partnership; living separately: married or in a partnership), and labor force participation (full-time employed; retired; other). Lifestyle-related factors included alcohol consumption (six categories from “never” to “daily”), smoking status (four categories from “never been a smoker” to “yes, daily”), and frequency of sports activity (consisting of five categories that ranged from an “absence of sports activity” to “regularly, more than 4 h a week).

The health-related factors consisted of self-rated health (from 1 = very poor to 5 = very good), and chronic conditions (i.e., a count score based on 14 chronic conditions such as stroke, cancer, or high blood pressure).

### 2.1 Statistical analysis

Characteristics of the sample are first described (also stratified by GD). Then, the prevalence rate of probable GD was calculated (among the total sample and also stratified by sociodemographic and lifestyle-related factors; p-values are based on Chi²-tests or oneway ANOVAs, as appropriate). Following this, the association between probable GD and the psychosocial outcomes was analyzed using linear regression (also stratified by age group and gender). As covariates, we included the following factors in regression analysis (please also see the section before): age, federal state, education, marital status, employment status, smoking status, alcohol consumption, sports activities, self-rated health, and the number of chronic illnesses.

With regard to standard errors, an HC3 bias correction (a jackknife estimator) (Davidson & MacKinnon, 1993) with Hansen adjustments (Hansen, 2025) was calculated.

In subsequent regression models, interaction terms were included to test whether the associations of GD with the psychosocial outcomes varied significantly by gender and age group.

Different effect sizes are presented (Cohen’s d for unadjusted comparisons and partial Eta^2^ for the regression results). Regarding specific effect sizes, Cohen’s d = 0.20 denotes a small effect size, d = 0.50 denotes a medium effect, and d = 0.80 denotes a large effect size (Cohen, 1988). For the partial eta^2^ values (Cohen, 1988): .01 = a small effect, .06 = a medium effect, and .14 = a large effect.

StataNow 19.5 MP-Parallel Edition (Stata Corp., College Station, Texas) was used for the statistical analysis. The significance level was set at p < 0.05.

## 3 Results

***Sample characteristics and bivariate analyses***.

Sample characteristics are shown in Table 1. In total, 59.2% of the study participants played computer games (whether offline or online) in the past 12 months.

**Table 1:** Sample characteristics (also stratified by gaming disorder).

Variables	Total sample (n = 3,270, 100%)	Not playing computer games (n = 1334, 40.8%)	Playing computer games, but without a probable gaming disorder (n = 1706, 52.2%)	Probable gaming disorder (n = 230, 7.0%)	P-value
Gender: N (%)					<0.001
Men	1614 (49.4)	571 (42.8)	888 (52.1)	155 (67.4)	
Women	1647 (50.4)	761 (57.0)	811 (47.5)	75 (32.6)	
Diverse	9 (0.3)	2 (0.1)	7 (0.4)	0 (0.0)	
Age: Mean (SD)	47.0 (15.3)	50.6 (14.9)	46.0 (14.9)	33.4 (10.9)	<0.001
Age group: N (%)					<0.001
18 to 29 years	582 (17.8)	168 (28.9)	301 (51.7)	113 (19.4)	
30 to 39 years	602 (18.4)	183 (30.4)	354 (58.8)	65 (10.8)	
40 to 49 years	553 (16.9)	211 (38.2)	314 (56.8)	28 (5.1)	
50 to 59 years	721 (22.0)	336 (46.6)	366 (50.8)	19 (2.6)	
60 years and older	812 (24.8)	436 (53.7)	371 (45.7)	5 (0.6)	
Marital status: N (%)					<0.001
Single	908 (27.8)	331 (24.8)	479 (28.1)	98 (42.6)	
Divorced	277 (8.5)	130 (9.7)	138 (8.1)	9 (3.9)	
Widowed	103 (3.1)	51 (3.8)	49 (2.9)	3 (1.3)	
Married, cohabiting with spouse	1867 (57.1)	771 (57.8)	981 (57.5)	115 (50.0)	
Married, not cohabiting with spouse	115 (3.5)	51 (3.8)	59 (3.5)	5 (2.2)	<0.001
Education: N (%)					<0.001
Low	337 (10.3)	127 (9.5)	169 (9.9)	41 (17.8)	
Medium	1552 (47.5)	626 (46.9)	838 (49.1)	88 (38.3)	
High	1381 (42.2)	581 (43.6)	699 (41.0)	101 (43.9)	
Employment status: N (%)					<0.001
Full-time employed	1629 (49.8)	593 (44.5)	893 (52.3)	143 (62.2)	
Retired	649 (19.8)	346 (25.9)	294 (17.2)	9 (3.9)	
Other	992 (30.3)	395 (29.6)	519 (30.4)	78 (33.9)	
Smoking status: N (%)					<0.001
Yes, daily	737 (22.5)	265 (19.9)	403 (23.6)	69 (30.0)	
Yes, sometimes	347 (10.6)	84 (6.3)	208 (12.2)	55 (23.9)	
No, not anymore	891 (27.2)	374 (28.0)	476 (27.9)	41 (17.8)	
Never smoker	1295 (39.6)	611 (45.8)	619 (36.3)	65 (28.3)	
Alcohol consumption: N (%)					<0.001
Daily	185 (5.7)	73 (5.5)	86 (5.0)	26 (11.3)	
Several times a week	560 (17.1)	195 (14.6)	304 (17.8)	61 (26.5)	
Once a week	578 (17.7)	229 (17.2)	301 (17.6)	48 (20.9)	
1–3 times a month	558 (17.1)	201 (15.1)	326 (19.1)	31 (13.5)	
Less often	782 (23.9)	347 (26.0)	400 (23.4)	35 (15.2)	
Never	607 (18.6)	289 (21.7)	289 (16.9)	29 (12.6)	
Sports activity: N (%)					<0.001
No sports activity	843 (25.8)	380 (28.5)	434 (25.4)	29 (12.6)	
Less than one hour a week	605 (18.5)	238 (17.8)	324 (19.0)	43 (18.7)	
Regularly, 1–2 h a week	813 (24.9)	311 (23.3)	419 (24.6)	83 (36.1)	
Regularly, 2–4 h a week	550 (16.8)	211 (15.8)	301 (17.6)	38 (16.5)	
Regularly, more than 4 h a week	459 (14.0)	194 (14.5)	228 (13.4)	37 (16.1)	
Self-rated health: Mean (SD)	3.6 (0.8)	3.5 (0.8)	3.6 (0.8)	3.8 (0.9)	<0.001
Count of chronic conditions: Mean (SD)	1.5 (1.6)	1.5 (1.6)	1.5 (1.6)	1.5 (1.7)	0.88
Depressive symptoms: Mean (SD)	7.0 (5.7)	5.8 (5.3)	7.1 (5.4)	13.3 (5.7)	<0.001
Anxiety symptoms: Mean (SD)	5.6 (5.1)	4.7 (4.8)	5.6 (5.0)	10.6 (4.7)	<0.001
Loneliness: Mean (SD)	3.3 (2.0)	3.1 (2.1)	3.4 (2.0)	4.3 (1.4)	<0.001
Social isolation: Mean (SD)	2.0 (0.8)	1.8 (0.8)	2.0 (0.8)	2.7 (0.7)	<0.001
Social withdrawal: Mean (SD)	38.0 (17.3)	36.0 (17.5)	38.1 (17.1)	49.3 (12.6)	<0.001
Life satisfaction: Mean (SD)	21.5 (6.4)	22.3 (6.2)	21.0 (6.4)	20.5 (6.9)	<0.001

Among individuals who played computer games in the past 12 months, 11.9% had a probable GD. Having probable GD was significantly associated with nearly all of the outcome variables (except for the number of chronic conditions). For example, when comparing individuals not playing computer games past 12 months and those with a probable GD, small (d = 0.29, 95% CI: 0.15 to 0.43, with life satisfaction), medium (d = -0.60, 95% CI: −0.74 to −0.46, with loneliness), and large effect sizes (d = -0.79, 95% CI: −0.94 to −0.65, with social withdrawal; d = -1.15, 95% CI: −1.30 to −1.01, with perceived social isolation; d = –1.24, 95% CI: −1.39 to −1.10, with anxiety symptoms; d = -1.38, 95% CI: −1.53 to −1.23, with depressive symptoms) were observed. In contrast, when individuals not playing computer games were compared with those playing computer games (but without probable GD), there were only small differences (in absolute terms: ranging from d = 0.11 to 0.23) for the psychosocial outcomes.

***Prevalence of probable gaming disorder***.

The prevalence of probable GD is shown in Table 2. Based on the total sample, 7.0% had a probable GD (as mentioned above: based on individuals who played computer games, 11.9% had a probable GD). The prevalence varied greatly by age group (e.g., 18 to 29 years: 19.4%; 30 to 39 years: 10.8%, 40 to 49 years: 5.1%), smoking status, and alcohol consumption (see Table 2).

**Table 2:** Prevalence of probable gaming disorder (among the total sample N = 3,270 and also stratified by sociodemographic and lifestyle-related factors).

	Gaming disorder		
	Not playing computer games n (%)	Playing computer games, but without a probable gaming disorder n (%)	Probable gaming disorder n (%)
Total sample	1334 (40.8)	1706 (52.2)	230 (7.0)
Gender			
Male	571 (35.4)	888 (55.0)	155 (9.6)
Female	761 (46.2)	811 (49.2)	75 (4.6)
Diverse	2 (22.2)	7 (77.8)	0 (0.0)
Age group			
18 to 29 years	168 (28.9)	301 (51.7)	113 (19.4)
30 to 39 years	183 (30.4)	354 (58.8)	65 (10.8)
40 to 49 years	211 (38.2)	314 (56.8)	28 (5.1)
50 to 59 years	336 (46.6)	366 (50.8)	19 (2.6)
60 years and older	436 (53.7)	371 (45.7)	5 (0.6)
Marital status			
Single	331 (36.5)	479 (52.8)	98 (10.8)
Divorced	130 (46.9)	138 (49.8)	9 (3.2)
Widowed	51 (49.5)	49 (47.6)	3 (2.9)
Living together: Married/Partnership	771 (41.3)	981 (52.5)	115 (6.2)
Living separated: Married/Partnership	51 (44.3)	59 (51.3)	5 (4.3)
Education			
Low	127 (37.7)	169 (50.1)	41 (12.2)
Medium	626 (40.3)	838 (54.0)	88 (5.7)
High	581 (42.1)	699 (50.6)	101 (7.3)
Employment status			
Full-time employed	593 (36.4)	893 (54.8)	143 (8.8)
Retired	346 (53.3)	294 (45.3)	9 (1.4)
Other	395 (39.8)	519 (52.3)	78 (7.9)
Smoking behavior			
Yes, daily	265 (36.0)	403 (54.7)	69 (9.4)
Yes, occasionally	84 (24.2)	208 (59.9)	55 (15.9)
No, not anymore	374 (42.0)	476 (53.4)	41 (4.6)
No, never	611 (47.2)	619 (47.8)	65 (5.0)
Alcohol consumption			
Daily	73 (39.5)	86 (46.5)	26 (14.1)
Several times per week	195 (34.8)	304 (54.3)	61 (10.9)
Once per week	229 (39.6)	301 (52.1)	48 (8.3)
1–3 times per month	201 (36.0)	326 (58.4)	31 (5.6)
Less often	347 (44.4)	400 (51.2)	35 (4.5)
Never	289 (47.6)	289 (47.6)	29 (4.8)
Frequency of sports activity			
Never			
Less than 1 h per week	380 (45.1)	434 (51.5)	29 (3.4)
Regularly, 1–2 h per week	238 (39.3)	324 (53.6)	43 (7.1)
Regularly, 2–4 h per week	311 (38.3)	419 (51.5)	83 (10.2)
Regularly, more than 4 h per week	211 (38.4)	301 (54.7)	38 (6.9)

***Regression analysis***.

The results of the linear regression analyses are presented in Table 3 (for the total sample), Table 4 (for men), and Table 5 (for women). Age-stratified findings can be found in Supplementary Tables 1 to 4.

**Table 3:** Association of playing computer games and psychosocial outcomes among the total sample. Results of linear regression analyses.

Probable gaming disorder	Depressive symptoms	Anxiety symptoms	Loneliness	Perceived social isolation	Social withdrawal	Life satisfaction
Not playing computer games	Reference category	Reference category	Reference category	Reference category	Reference category	Reference category
Playing computer games, but without a probable gaming disorder	0.61***	0.35*	0.11	0.07**	1.60**	−1.11***
	(0.28–––0.94)	(0.03–––0.66)	(−0.03–––0.25)	(0.02–––0.13)	(0.43–––2.77)	(−1.51 − −0.71)
Probable gaming disorder	5.47***	4.28***	0.89***	0.72***	11.17***	−1.77***
	(4.67–––6.28)	(3.59–––4.96)	(0.65–––1.13)	(0.60–––0.84)	(9.06–––13.28)	(−2.68 − −0.85)
Covariates	✓	✓	✓	✓	✓	✓
R^2^	0.38	0.32	0.16	0.22	0.21	0.29
Observations	3,270	3,270	3,270	3,270	3,270	3,270

Unstandardized beta-coefficients are shown (95% CI in parentheses); *** p < 0.001, ** p < 0.01, * p < 0.05, + p < 0.10; sociodemographic covariates include age, gender, federal state, education, marital status, and employment status; lifestyle-related covariates include smoking status, alcohol consumption, and sports activities; health-related covariates include self-rated health and the number of chronic illnesses.

**Table 4:** Association of playing computer games and psychosocial outcomes among men. Results of linear regression analyses.

Probable gaming disorder	Depressive symptoms	Anxiety symptoms	Loneliness	Perceived social isolation	Social withdrawal	Life satisfaction
Not playing computer games	Reference category	Reference category	Reference category	Reference category	Reference category	Reference category
Playing computer games, but without a probable gaming disorder	0.52*	0.39+	−0.04	0.06	0.62	−0.72*
	(0.03–––1.01)	(−0.06–––0.83)	(−0.25–––0.16)	(−0.02–––0.14)	(−1.15–––2.40)	(−1.32 − −0.12)
Probable gaming disorder	5.68***	4.82***	0.81***	0.76***	10.17***	−1.71**
	(4.60–––6.76)	(3.92–––5.72)	(0.49–––1.13)	(0.61–––0.92)	(7.29–––13.04)	(−2.98 − −0.43)
Covariates	✓	✓	✓	✓	✓	✓
R^2^	0.38	0.32	0.16	0.25	0.22	0.30
Observations	1,614	1,614	1,614	1,614	1,614	1,614

Unstandardized beta-coefficients are shown (95% CI in parentheses); *** p < 0.001, ** p < 0.01, * p < 0.05, + p < 0.10; sociodemographic covariates include age, federal state, education, marital status, and employment status; lifestyle-related covariates include smoking status, alcohol consumption, and sports activities; health-related covariates include self-rated health and the number of chronic illnesses.

**Table 5:** Association of playing computer games and psychosocial outcomes among women. Results of linear regression analyses.

Probable gaming disorder	Depressive symptoms	Anxiety symptoms	Loneliness	Perceived social isolation	Social withdrawal	Life satisfaction
Not playing computer games	Reference category	Reference category	Reference category	Reference category	Reference category	Reference category
Playing computer games, but without a probable gaming disorder	0.68**	0.31	0.21*	0.07+	2.10*	−1.41***
	(0.24–––1.13)	(−0.14–––0.75)	(0.01–––0.41)	(−0.01–––0.15)	(0.48–––3.72)	(−1.96 − −0.86)
Probable gaming disorder	5.01***	3.48***	1.02***	0.64***	12.40***	−1.79**
	(3.82–––6.20)	(2.41–––4.56)	(0.64–––1.41)	(0.45–––0.83)	(9.10–––15.69)	(−3.06 − −0.51)
Covariates	✓	✓	✓	✓	✓	✓
R^2^	0.40	0.32	0.17	0.22	0.21	0.30
Observations	1,647	1,647	1,647	1,647	1,647	1,647

Unstandardized beta-coefficients are shown (95% CI in parentheses); *** p < 0.001, ** p < 0.01, * p < 0.05, + p < 0.10; sociodemographic covariates include age, federal state, education, marital status, and employment status; lifestyle-related covariates include smoking status, alcohol consumption, and sports activities; health-related covariates include self-rated health and the number of chronic illnesses.

Among the total sample, in fully adjusted regression analyses individuals playing computer games, but who did not have a probable GD had significantly poorer psychosocial outcomes (except for loneliness) compared to individuals not playing computer games. Further, individuals with a probable GD had markedly poorer psychosocial outcomes compared to individuals not playing computer games. Partial eta^2^-values for the GD variable ranged from 0.01 (for life satisfaction; loneliness), to over 0.03 (for social withdrawal), and over 0.05 (for perceived social isolation; anxiety symptoms) to 0.07 (for depressive symptoms).

Among men (Table 4), respondents playing computer games, but without a probable GD, had significantly more depressive symptoms and lower life satisfaction compared to individuals not playing computer games. Men with a probable GD had markedly poorer psychosocial outcomes for all of the variables compared to men not playing computer games.

Among women (Table 5), respondents playing computer games, but without a probable GD had significantly more depressive symptoms, higher loneliness levels, higher levels of social withdrawal, and lower life satisfaction levels. Furthermore, women with a probable GD had markedly poorer psychosocial outcomes for all of the variables compared to women not playing computer games. Most of the interaction terms (i.e., gender x GD) did not reach statistical significance (except for the terms for gender x respondents playing computer games, but without a probable GD vs. respondents not playing computer games; with loneliness and life satisfaction as outcomes; both significant at the p < 0.05 level). When compared to the findings for the total sample, the age-stratified findings revealed analogous results for each age group. The detailed results can be found in Supplementary Tables 1 to 4. Nearly all interaction terms (age group x GD) did not reach statistical significance.

## 4 Discussion

The aim of this study was to identify the prevalence of GD and to investigate its association with a variety of psychosocial outcomes using data from the general adult population in Germany. Our key findings: A significant proportion of individuals (7.0% of the entire sample) had a probable GD. Regression analyses showed that GD was significantly associated with poorer psychosocial outcomes. Similar associations were observed for both genders and different age groups. By using data from the general adult population in Germany (including young, middle-aged and older adults), this study builds on and extends the results of prior research that has been mainly based on samples of adolescents and young adults. Furthermore, while the previous research among adults occurred a number of years ago, this study used very recent data. Moreover, when examining the association of probable GD with various psychosocial outcomes, psychometrically sound tools were used.

An earlier meta-analysis showed somewhat lower prevalence rates for GD (Kim et al., 2022). However, a German study that included individuals aged 12 to 25 years and used the same tool as in the current study found a prevalence of 5.7% (in a sensitivity analysis with multiple imputation: 7.0%) in September 2017 (Wartberg et al., 2017). Given the fact that multiple crises, taking different forms, have occurred in recent years and the use of mobile devices is now widespread, it appears plausible that GD prevalence rates would have increased in the most recent period (in our sample, the prevalence rate was 19.4% among individuals aged 18 to 29 years). It is also of note that while the prevalence of GD was, as expected, relatively high, particularly among younger adults, we found that even among individuals aged 40–49 years, 5.1% could be classified as having probable GD (with the figure being 10.8% among those aged 30–39 years). Our findings thus indicate that GD is a challenge not only among adolescents and young adults, but also among middle-aged adults.

Previous studies, which have been mostly undertaken among adolescents or young adults, showed comparable associations between GD and psychosocial outcomes. For example, research has shown that GD can contribute to low social support and poor life satisfaction among individuals aged 17 to 21 years (Teng et al., 2020). Another study undertaken primarily among students from one U.S. university found that GD is associated with more depressive and anxiety symptoms as well as lower life satisfaction (Bargeron & Hormes, 2017). Furthermore, a study among Dutch adolescents showed a clear association between pathological gaming and loneliness (Lemmens et al., 2011). Additionally, a scoping review demonstrated an association between GD and social isolation (Richard et al., 2020), while a preliminary study found a link between GD and social withdrawal based on young adult players of online games (Stavropoulos et al., 2019) (Shah et al., 2024).

In this study we found significant, but only small differences in the associations with the psychosocial outcomes between individuals who did not and who played computer games (but without probable GD). This suggests that playing such games (in a non-pathological manner) might be associated with only small negative side effects (e.g., in terms of such things as the quality of the relationships with friends and relatives). More importantly, our results showed that probable GD was moderately to strongly associated with a number of detrimental psychosocial outcomes. In relation to this, it is possible that individuals with problematic gaming habits might more frequently lose control over their computer gaming behavior (Paulus et al., 2018). They may prioritize playing computer games over other responsibilities, such as vocational training, studies, work, family duties, or leisure activities (Ko & Yen, 2020). For instance, hobbies might be neglected in favor of playing computer games. Moreover, individuals with GD may continue to play despite experiencing negative consequences such as insomnia, conflicts with family and friends, poor job performance, unhealthy eating habits, vision issues, or pain in their wrists or hands (Babamiri et al., 2018, Bansal and Kranti, 2022, Che Mokhtar and McGee, 2025, Mylona et al., 2020). Excessive gaming may also lead to further withdrawal from society (Stavropoulos et al., 2019). Such withdrawal can make it more difficult to stay connected with friends in real life, with some individuals possibly becoming alienated from friends, relatives, and partners (Kosa et al., 2025). Overall, social life could suffer significantly as a result. Moreover, stress (e.g., at work) and other life events could also be more difficult to cope with for such individuals with GD. Such factors might help explain why individuals with probable GD reported poorer psychosocial outcomes in this study.

It should be noted that when comparing women not playing computer games and women playing computer games (but without probable GD), the latter group reported significantly higher social withdrawal and loneliness levels, whilst such a result was not observed among men. It can be speculated that, as playing computer games per se might be perceived as unusual for adult women, it may be accompanied by a certain stigma (Kuss et al., 2022) and that this might lead to female gamers (without GD) starting to withdraw somewhat from society, and that this may negatively affect the quality of their relationships (McLean & Griffiths, 2019). An important task for future research will be to investigate such associations in more detail (e.g., based on qualitative approaches).

This study has both strengths and weaknesses. Data came from a large, quota-based sample (reflecting the general adult population in Germany in terms of gender x age group, and federal state). Psychometrically sound tools were used to quantify the psychosocial outcomes and probable GD. However, future research based on a clinical diagnosis of GD is recommended. Clarifying the directionality of the observed associations is challenging due to the cross-sectional nature of our data (e.g., social withdrawal may also contribute to future changes in GD). In addition, as the data came from an online sample this may have resulted in different forms of bias occurring, although it is worth noting that almost every adult in this age group has access to the internet in Germany (German Federal Statistical Office, 2023). Some covariates such as lifestyle-related factors (e.g., alcohol consumption or frequency of sports activity) were only roughly operationalized. Future studies should investigate this in greater depth using validated instruments.

In conclusion, this study showed that individuals with probable GD are at high risk of poor psychosocial outcomes among the general adult population (and that this finding applies to both women and men as well as to different age groups). Future studies focusing on the mechanisms underlying these associations are warranted. Additionally, future longitudinal studies and cross-country comparisons are also recommended.

Funding.

This research did not receive any specific grant from funding agencies in the public, commercial, or not-for-profit sectors.

Statement of ethical approval.

Approval for the study was provided by the Local Psychological Ethics Committee of the Center for Psychosocial Medicine of the University Medical Center Hamburg-Eppendorf (number: LPEK-0849). Our study is in accordance with the ethical standards laid down in the 1964 Declaration of Helsinki and its later amendments.

## CRediT authorship contribution statement

**André Hajek:** Writing – review & editing, Writing – original draft, Visualization, Project administration, Methodology, Formal analysis, Data curation, Conceptualization. **Andrew Stickley:** Writing – review & editing, Visualization, Conceptualization. **Karl Peltzer:** Writing – review & editing, Visualization, Conceptualization. **Supa Pengpid:** Writing – review & editing, Visualization, Conceptualization. **Hans-Helmut König:** Writing – review & editing, Visualization, Supervision, Resources, Conceptualization.

## Declaration of competing interest

The authors declare that they have no known competing financial interests or personal relationships that could have appeared to influence the work reported in this paper.

## Data Availability

The data that has been used is confidential.
