# Supplementary material for: Gaming disorder: Prevalence and association with psychosocial outcomes in the German general adult population
Source: Addict Behav Rep. 2026 Feb 21;23:100682. doi: 10.1016/j.abrep.2026.100682 (PMC12955077; doi:10.1016/j.abrep.2026.100682)
Supplement: Supplementary Data 1 [file mmc1.docx]

Supplementary Table 1. Association of playing computer games and psychosocial outcomes among individuals aged 18 to 29 years. Results of linear regression analyses

| Independent variables | Depressive symptoms | Anxiety symptoms | Loneliness | Perceived social isolation | Social withdrawal | Life satisfaction |
| --- | --- | --- | --- | --- | --- | --- |
|  |  |  |  |  |  |  |
| Not playing computer games | Reference category | Reference category | Reference category | Reference category | Reference category | Reference category |
|  |  |  |  |  |  |  |
| Playing computer games, but without a probable gaming disorder | 0.86+ | 0.42 | 0.20 | 0.06 | 0.95 | -1.26* |
|  | (-0.15 - 1.87) | (-0.52 - 1.35) | (-0.18 - 0.58) | (-0.09 - 0.22) | (-2.28 - 4.19) | (-2.28 - -0.24) |
|  |  |  |  |  |  |  |
| Probable gaming disorder | 4.34*** | 3.09*** | 0.87*** | 0.56*** | 7.61*** | -0.69 |
|  | (2.83 - 5.86) | (1.84 - 4.34) | (0.41 - 1.33) | (0.34 - 0.78) | (3.49 - 11.72) | (-2.15 - 0.76) |
|  |  |  |  |  |  |  |
| Covariates | ✓ | ✓ | ✓ | ✓ | ✓ | ✓ |
|  |  |  |  |  |  |  |
| R² | 0.31 | 0.26 | 0.17 | 0.21 | 0.19 | 0.35 |
| Observations | 582 | 582 | 582 | 582 | 582 | 582 |

Unstandardized beta-coefficients are shown (95% CI in parentheses); *** p<0.001, ** p<0.01, * p<0.05, + p<0.10; sociodemographic covariates include age, gender, federal state, education, marital status, and employment status; lifestyle-related covariates include smoking status, alcohol consumption, and sports activities; health-related covariates include self-rated health and the number of chronic illnesses.

Supplementary Table 2. Association of playing computer games and psychosocial outcomes among individuals aged 30 to 39 years. Results of linear regression analyses

| Independent variables | Depressive symptoms | Anxiety symptoms | Loneliness | Perceived social isolation | Social withdrawal | Life satisfaction |
| --- | --- | --- | --- | --- | --- | --- |
|  |  |  |  |  |  |  |
| Not playing computer games | Reference category | Reference category | Reference category | Reference category | Reference category | Reference category |
|  |  |  |  |  |  |  |
| Playing computer games, but without a probable gaming disorder | 0.57 | 0.04 | -0.30+ | 0.08 | 2.33+ | -1.06* |
|  | (-0.36 - 1.50) | (-0.78 - 0.86) | (-0.65 - 0.04) | (-0.07 - 0.22) | (-0.44 - 5.10) | (-2.07 - -0.05) |
|  |  |  |  |  |  |  |
| Probable gaming disorder | 6.26*** | 4.31*** | 0.67* | 0.66*** | 12.61*** | -3.01** |
|  | (4.53 - 7.99) | (2.86 - 5.75) | (0.15 - 1.19) | (0.41 - 0.92) | (8.54 - 16.67) | (-4.81 - -1.21) |
|  |  |  |  |  |  |  |
| Covariates | ✓ | ✓ | ✓ | ✓ | ✓ | ✓ |
|  |  |  |  |  |  |  |
| R² | 0.36 | 0.32 | 0.24 | 0.21 | 0.29 | 0.28 |
| Observations | 602 | 602 | 602 | 602 | 602 | 602 |

Unstandardized beta-coefficients are shown (95% CI in parentheses); *** p<0.001, ** p<0.01, * p<0.05, + p<0.10; sociodemographic covariates include age, gender, federal state, education, marital status, and employment status; lifestyle-related covariates include smoking status, alcohol consumption, and sports activities; health-related covariates include self-rated health and the number of chronic illnesses.

Supplementary Table 3. Association of playing computer games and psychosocial outcomes among individuals aged 40 to 49 years. Results of linear regression analyses

| Independent variables | Depressive symptoms | Anxiety symptoms | Loneliness | Perceived social isolation | Social withdrawal | Life satisfaction |
| --- | --- | --- | --- | --- | --- | --- |
|  |  |  |  |  |  |  |
| Not playing computer games | Reference category | Reference category | Reference category | Reference category | Reference category | Reference category |
|  |  |  |  |  |  |  |
| Playing computer games, but without a probable gaming disorder | 0.53 | 0.91* | 0.23 | 0.04 | 1.66 | -1.04* |
|  | (-0.32 - 1.38) | (0.09 - 1.73) | (-0.13 - 0.59) | (-0.11 - 0.18) | (-1.45 - 4.78) | (-2.04 - -0.04) |
|  |  |  |  |  |  |  |
| Probable gaming disorder | 5.52*** | 5.22*** | 1.17*** | 0.75*** | 12.52*** | -1.59 |
|  | (3.62 - 7.41) | (3.47 - 6.96) | (0.53 - 1.81) | (0.43 - 1.06) | (6.94 - 18.10) | (-4.37 - 1.18) |
|  |  |  |  |  |  |  |
| Covariates | ✓ | ✓ | ✓ | ✓ | ✓ | ✓ |
|  |  |  |  |  |  |  |
| R² | 0.36 | 0.30 | 0.20 | 0.22 | 0.26 | 0.37 |
| Observations | 553 | 553 | 553 | 553 | 553 | 553 |

Unstandardized beta-coefficients are shown (95% CI in parentheses); *** p<0.001, ** p<0.01, * p<0.05, + p<0.10; sociodemographic covariates include age, gender, federal state, education, marital status, and employment status; lifestyle-related covariates include smoking status, alcohol consumption, and sports activities; health-related covariates include self-rated health and the number of chronic illnesses.

Supplementary Table 4. Association of playing computer games and psychosocial outcomes among individuals aged 50 to 74 years. Results of linear regression analyses

| Independent variables | Depressive symptoms | Anxiety symptoms | Loneliness | Perceived social isolation | Social withdrawal | Life satisfaction |
| --- | --- | --- | --- | --- | --- | --- |
|  |  |  |  |  |  |  |
| Not playing computer games | Reference category | Reference category | Reference category | Reference category | Reference category | Reference category |
|  |  |  |  |  |  |  |
| Playing computer games, but without a probable gaming disorder | 0.63** | 0.25 | 0.18+ | 0.08* | 1.26 | -1.08*** |
|  | (0.20 - 1.06) | (-0.17 - 0.68) | (-0.02 - 0.38) | (0.00 - 0.16) | (-0.41 - 2.94) | (-1.65 - -0.50) |
|  |  |  |  |  |  |  |
| Probable gaming disorder | 6.00*** | 5.16*** | 1.20** | 1.01*** | 14.15*** | -2.72 |
|  | (3.92 - 8.09) | (3.52 - 6.80) | (0.47 - 1.93) | (0.71 - 1.31) | (8.57 - 19.73) | (-6.05 - 0.62) |
|  |  |  |  |  |  |  |
| Covariates | ✓ | ✓ | ✓ | ✓ | ✓ | ✓ |
|  |  |  |  |  |  |  |
| R² | 0.39 | 0.30 | 0.17 | 0.19 | 0.23 | 0.31 |
| Observations | 1,533 | 1,533 | 1,533 | 1,533 | 1,533 | 1,533 |

Unstandardized beta-coefficients are shown (95% CI in parentheses); *** p<0.001, ** p<0.01, * p<0.05, + p<0.10; sociodemographic covariates include age, gender, federal state, education, marital status, and employment status; lifestyle-related covariates include smoking status, alcohol consumption, and sports activities; health-related covariates include self-rated health and the number of chronic illnesses.
